# Supplementary material for: FtsZ induces membrane deformations via torsional stress upon GTP hydrolysis
Source: Nat Commun. 2021 Jun 3;12:3310. doi: 10.1038/s41467-021-23387-3 (PMC8175707; doi:10.1038/s41467-021-23387-3)
Supplement: Supplementary file 1 — Supplementary Information [file 41467_2021_23387_MOESM1_ESM.pdf]

FtsZ induces membrane deformations via torsional stress upon GTP hydrolysis.

Authors: Diego A. Ramirez-Diaz[1,2], Adrian Merino-Salomon[1,3], Fabian Meyer[4], Michael Heymann[1,5], German Rivas[6], Marc Bramkamp[4] & Petra Schwille[1*].

Supplementary Materials

This pdf contains:

Fig. S1 to S4

Supplementary Table 1-2 (Cloning vectors and primers)

Fig. S1.

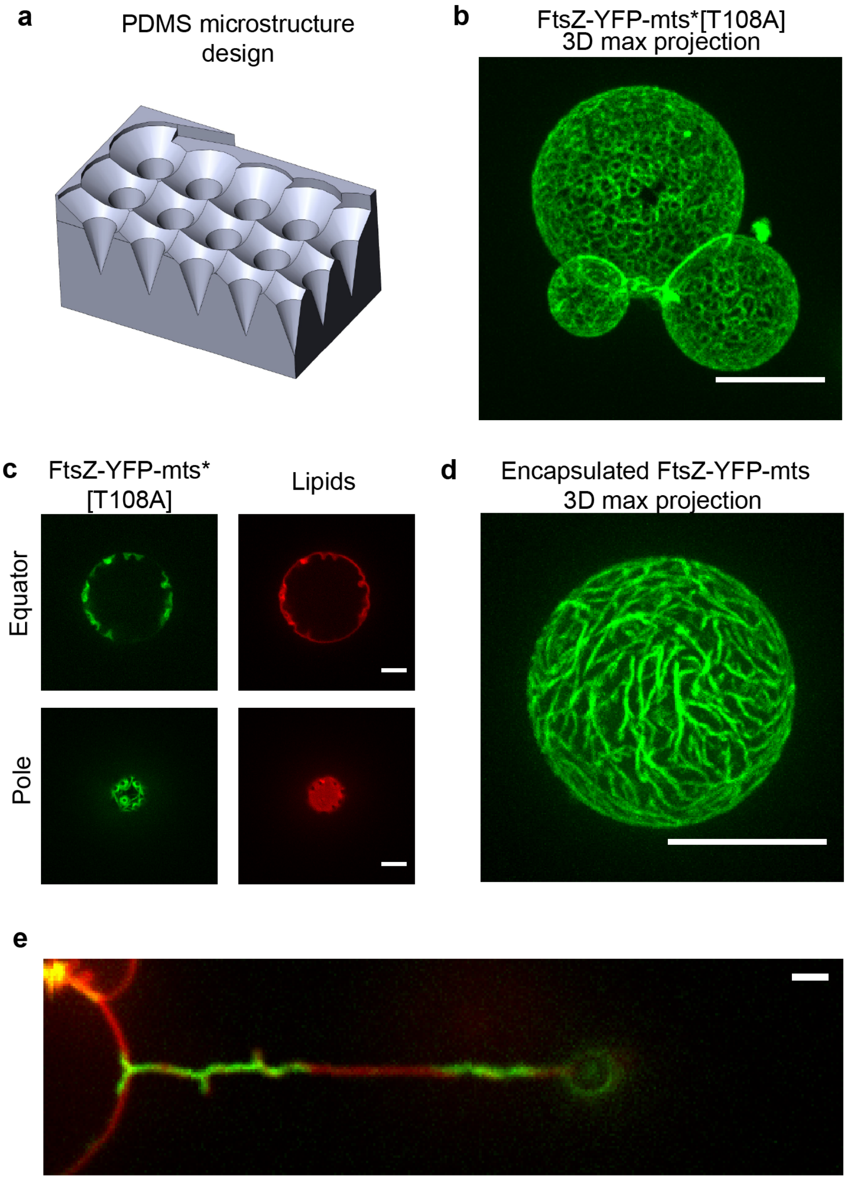

Design of microstructure, FtsZ-YFP-mts*[T108A] deformations, high density encapsulated FtsZ-YFP-mts and FtsZ-YFP-mts supercoiling, a) 3D sketch of the PMDS microstructure with inwards cone-like shapes. b) FtsZ-YFP-mts*[T108A] also self-assembled into ring-like structures on GUVs (GUVs:N>20) (Scale bar = 10 µm). c) After deflation, FtsZ-YFP-mts*[T108A] induced inwards conical deformations emerging from before mentioned rings (GUVs:N>20) (Scale bar = 2 µm). d) By encapsulating FtsZ-YFP-mts in GUVs, Mg^{+2} and GTP content-conditions were fine-tuned to obtain either ring structures (Fig. 5) or nematic phases at a higher membrane protein density (GUVs:N>10) (Scale bar = 10 µm). e) FtsZ-YFP-

mts torsion over lipid tubes can be found in (less frequent) experiments displaying plectonic/supercoiled regions (N=2). (Scale bar = 2 µm).

Fig. S2.

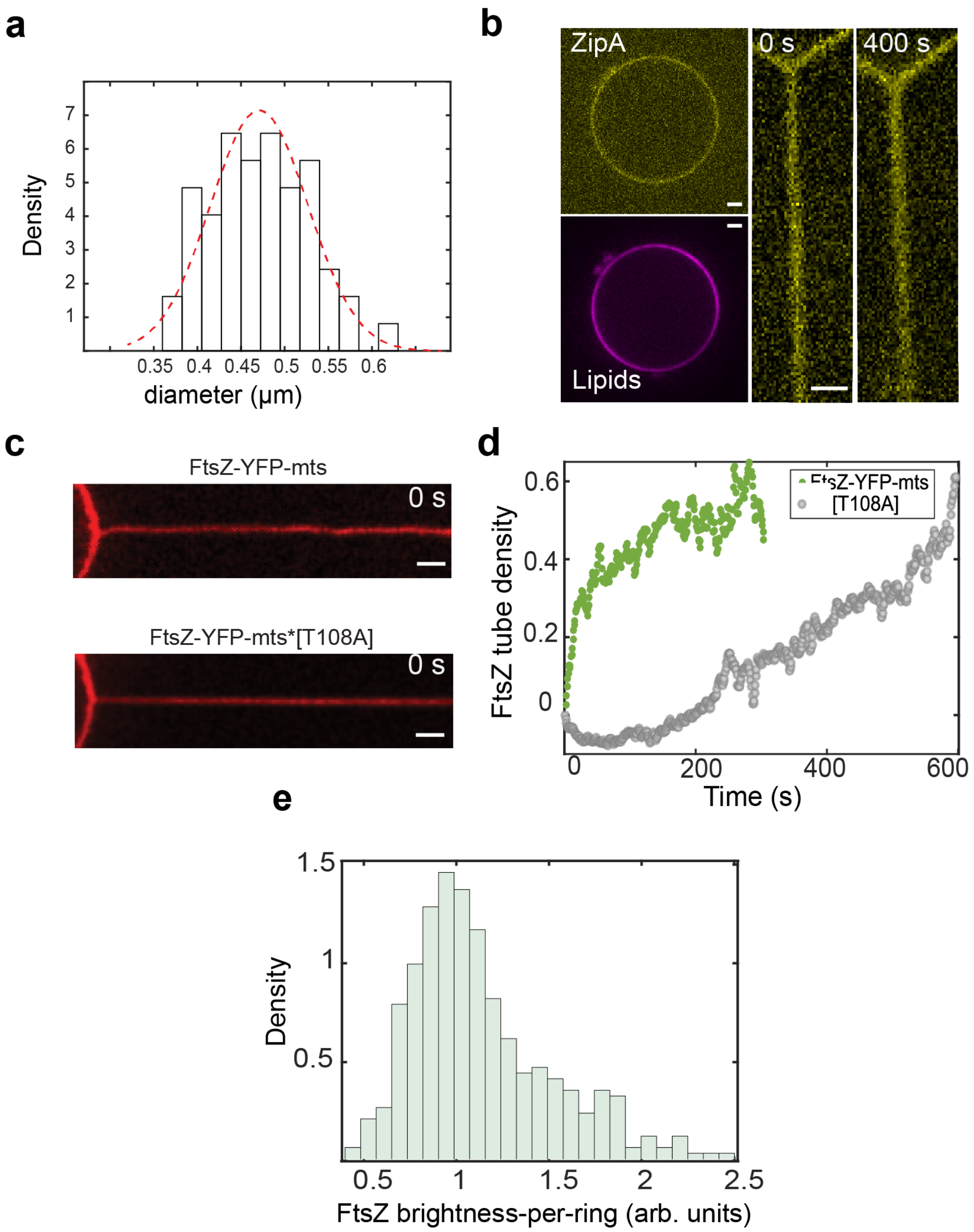

Tube diameter distribution, ZipA control experiments and ring-unit-brightness distribution. a) The diameter distribution (N=55) showed a Gaussian distribution with a mean of 0.47 µm. This implied that membrane tensions equivalent to this mean (± std) were highly

frequent despite of precise control on the vesicle membrane tension. b) Vesicles decorated with ZipA and imaged under deflation conditions exhibited no deformations (GUVs:N=14). In addition, we examined lipid tubes only coated with siZipA-Alexa 488. No deformations (N=10) were observed in the range of 400-600 seconds. c) Similar initial lipid tube diameter (0.44 µm) for experiments shown in Figure 3 a-b d) For experiments shown in Fig. 3 a-b, FtsZ-YFP-mts entered rapidly to the lipid tube while the mutant without GTPase activity (slower). e). Distribution of FtsZ brightness-per-ring (N=412 analyzed rings). The distribution's mode value was chosen as the value for FtsZ brightness-per-ring. (Scale bar = 2 µm).

Fig. S3.

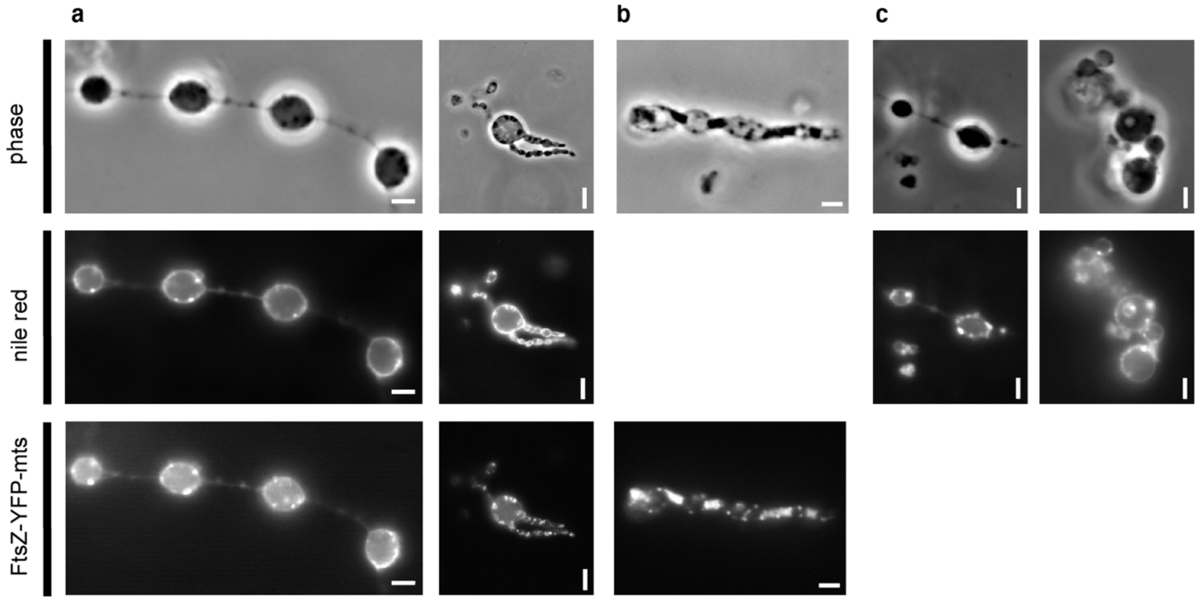

Observed phenotypes of *E. coli ftsZ-YFP-mts* after lysozyme treatment in sucrose-buffer. The first columns in a, b & b show a pearl necklace like appearance of the cells, while the second column in b might show an earlier stage of this chaining type of vesiculation. Nile red was used to prove that the observed vesicles and tubular connections are actually phospholipid-membranes. As a control unstained cells b) and stained cells c) were also imaged separately in

the respective channel. Example micrographs from (N=3) biological replicates. (scale bar = 2μm).

Fig. S4

a

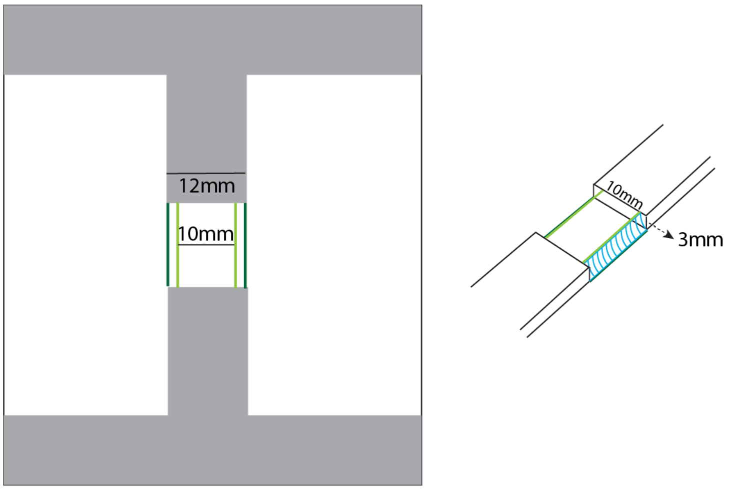

b

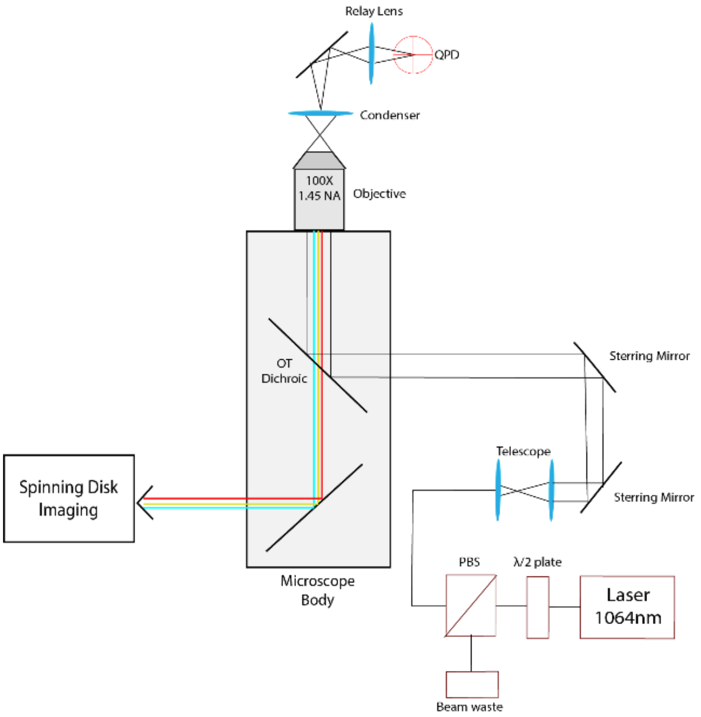

Drawing for the experiment chamber and optical trapping device a) Sketch of imaging chamber for deflated vesicle preparation. b) Optical tweezers setup layout.

Supplementary Table 1: Cloning Vectors

Vector	Protein	Source/reference
pET-11b	FtsZ-YFP-mts	gifted by Harold Erickson, Ref. 1
pET-11b	FtsZ-YFP-mts*[T108A]	Ref. 3
pET-28a	FtsZ-WT	Ref. 31
pET-15ZIP	sZipA	Ref. 32
pEKEx2	FtsZ-YFP-mts	This publication

Supplementary Table 2: Primers

Name	Sequence 5' – 3'
T108A_RV	GGTGGTGGTGCCGGTACAGGT
T108A_FW	ACCTGTACCGGCACCACCACC
sZipAI	CATATGGCTGCCGCGCG
sZipAII	ACCAGCCGTAAAGAACG
SalI-ftsZ	CATGTCGACATGTTTGAACCAATGGAACTTACC
SacI-mts	CATGAGCTCTTATCCTCCGAACAAGCG
