# Supplementary material for: FtsZ induces membrane deformations via torsional stress upon GTP hydrolysis
Source: Nat Commun. 2021 Jun 3;12:3310. doi: 10.1038/s41467-021-23387-3 (PMC8175707; doi:10.1038/s41467-021-23387-3)
Supplement: Supplementary file 3 — Description of Additional Supplementary Files [file 41467_2021_23387_MOESM3_ESM.docx]

**Description of Supplementary Files**

**File Name: Supplementary Data 1**

**Description:** Clockwise and counterclockwise FtsZ-YFP treadmilling inside PDMS cones.

**File Name: Supplementary Data 2**

**Description:** Soft-vesicle exhibiting inwards deformations induced by FtsZ-YFP-mts.

**File Name: Supplementary Data 3**

**Description:** After lipid tube pulling, FtsZ-YFP-mts induced coiling that resulted in the transformation of the lipid tube into a spring-like structure.

**File Name: Supplementary Data 4**

**Description:** After lipid tube pulling, FtsZ-YFP-mts*[T108A] induced helical transformations.

**File Name: Supplementary Data 5**

**Description:** Oscillation of GUV to measure the force response.

**File Name: Supplementary Data 6**

**Description:** Z-stacks of cell displaying vesiculation. Left. Phase. Right. FtsZ-YFP-mts.
